# The use of photoplethysmography for assessing hypertension

**DOI:** 10.1038/s41746-019-0136-7

**Published:** 2019-06-26

**Authors:** Mohamed Elgendi, Richard Fletcher, Yongbo Liang, Newton Howard, Nigel H. Lovell, Derek Abbott, Kenneth Lim, Rabab Ward

**Affiliations:** 1 School of Electrical and Computer Engineering, University of British Columbia, Vancouver, Canada; 2 Department of Obstetrics & Gynecology, University of British Columbia, Vancouver, Canada; 3 BC Children’s & Women’s Hospital, Vancouver, Canada; 4 D-Lab, Massachusetts Institute of Technology, Cambridge, MA USA; 5 Department of Psychiatry, University of Massachusetts Medical School, Worcester, MA USA; 6 Nuffield Department of Surgical Sciences, University of Oxford, Oxford, UK; 7 Howard Brain Sciences Foundation, Providence, Rhode Island USA; 8 Graduate School of Biomedical Engineering, UNSW Sydney, Sydney, NSW Australia; 9 School of Electrical and Electronic Engineering, The University of Adelaide, Adelaide, SA Australia; 10 Centre for Biomedical Engineering, The University of Adelaide, Adelaide, SA Australia

**Keywords:** Electrocardiography - EKG, Data integration, Statistical methods, Diagnostic markers, Predictive markers

## Abstract

The measurement of blood pressure (BP) is critical to the treatment and management of many medical conditions. High blood pressure is associated with many chronic disease conditions, and is a major source of mortality and morbidity around the world. For outpatient care as well as general health monitoring, there is great interest in being able to accurately and frequently measure BP outside of a clinical setting, using mobile or wearable devices. One possible solution is photoplethysmography (PPG), which is most commonly used in pulse oximetry in clinical settings for measuring oxygen saturation. PPG technology is becoming more readily available, inexpensive, convenient, and easily integrated into portable devices. Recent advances include the development of smartphones and wearable devices that collect pulse oximeter signals. In this article, we review (i) the state-of-the-art and the literature related to PPG signals collected by pulse oximeters, (ii) various theoretical approaches that have been adopted in PPG BP measurement studies, and (iii) the potential of PPG measurement devices as a wearable application. Past studies on changes in PPG signals and BP are highlighted, and the correlation between PPG signals and BP are discussed. We also review the combined use of features extracted from PPG and other physiological signals in estimating BP. Although the technology is not yet mature, it is anticipated that in the near future, accurate, continuous BP measurements may be available from mobile and wearable devices given their vast potential.

## Introduction

With the advancement of digital sensors, signal processing, machine-learning algorithms, and improved physiologic models, pulse waveform analysis using photophlethysmography (PPG) for the assessment of blood pressure (BP) has become more feasible.^1–4^ PPG signal measurements is not without its challenges; it requires noise elimination,^5–7^ multi-site measurement^8^, multi-photodectors development,^9^ event detection,^10^ event visualization,^11^ different models,^12^ and a thorough global health framework.^13^ Several disadvantages are associated with this method, including the need to conduct an individual calibration for each person, based on skin color and clinical factors, and the drift in calibration over short-time intervals.^14^

## Photoplethysmography

First explored in the 1930’s, PPG is a method for measuring the amount of light that is absorbed or reflected by blood vessels in living tissue. Since the amount of optical absorption or reflection depends on the amount of blood that is present in the optical path, the PPG signal is responsive to changes in the volume of the blood, rather than the pressure of the blood vessels. In other words, PPG detects the change of blood volume by the photoelectric technique, whether transmissive or reflective, to record the volume of blood in the sensor coverage area to form a PPG signal. Indeed, the sensor coverage area includes both veins and arteries, and numerous capillaries. Thus, the PPG signal is a complex mixture of the blood flow in veins and arteries of the cardiovascular circulatory system. A raw PPG signal generally includes pulsatile and non-pulsatile blood volume.^15^

The pulsatile component of a PPG signal is related to changes in blood volume inside the arteries and is synchronous with the heartbeat, whereas the non-pulsating component is a function of the basic blood volume, respiration, the sympathetic nervous system, and thermoregulation.^16^ In clinical practice, PPG is routinely used to monitor cardiac-induced blood volume changes in microvascular beds at peripheral body sites, such as the finger, forehead, earlobe, and toe.^17^ Since the maximum pulsatile component of reflected light occurs approximately in the range between 510 and 590 nm,^18^ the green (565 nm) or yellow (590 nm) light is generally used for reflective PPG sensors.^19^ However, the red (680 nm) or near-infrared (810 nm) light is generally used for transmissive PPG devices, with the infrared light having the deepest penetration.^20,21^ Given that the optical absorption of hemoglobin is a function of oxygenation and optical wavelength, the use of PPG at multiple wavelengths is also routinely used in pulse oximetry.

Green and red infrared light are often used to obtain PPG signals because of the difference in the wavelength; each light penetrates human tissue differently. Infrared light has the deepest penetration ability, and it can reflect the blood pulse from deep tissue. Therefore, it is used more. Red and infrared light can penetrate about 2.5 mm,^22^ while green light can penetrate less than 1 mm^22^ into tissue. Hence, the detection of blood pressure, atherosclerosis, blood sugar, and other physiological parameters uses the infrared light (deeper light penetration compared with the green light) to obtain PPG signals.

PPG technology thus represents a convenient and low-cost technology^23^ that can be applied to various aspects of cardiovascular monitoring, including the detection of blood oxygen saturation, heart rate, BP, cardiac output, respiration, arterial aging, endothelial function, microvascular blood flow, and autonomic function.^8^ Several different types of PPG waveforms have been observed and found to correlate with age and cardiovascular pathology.^24,25^ Since the volume and distension of the arteries can be related to the pressure in the arteries, the PPG signal produces pulse waveforms that are very similar to pressure waveforms generated by tonometry; however, PPG offers the added advantage that it can be measured continuously using miniature, inexpensive, and wearable optical electronics.

**Fig. 1:**
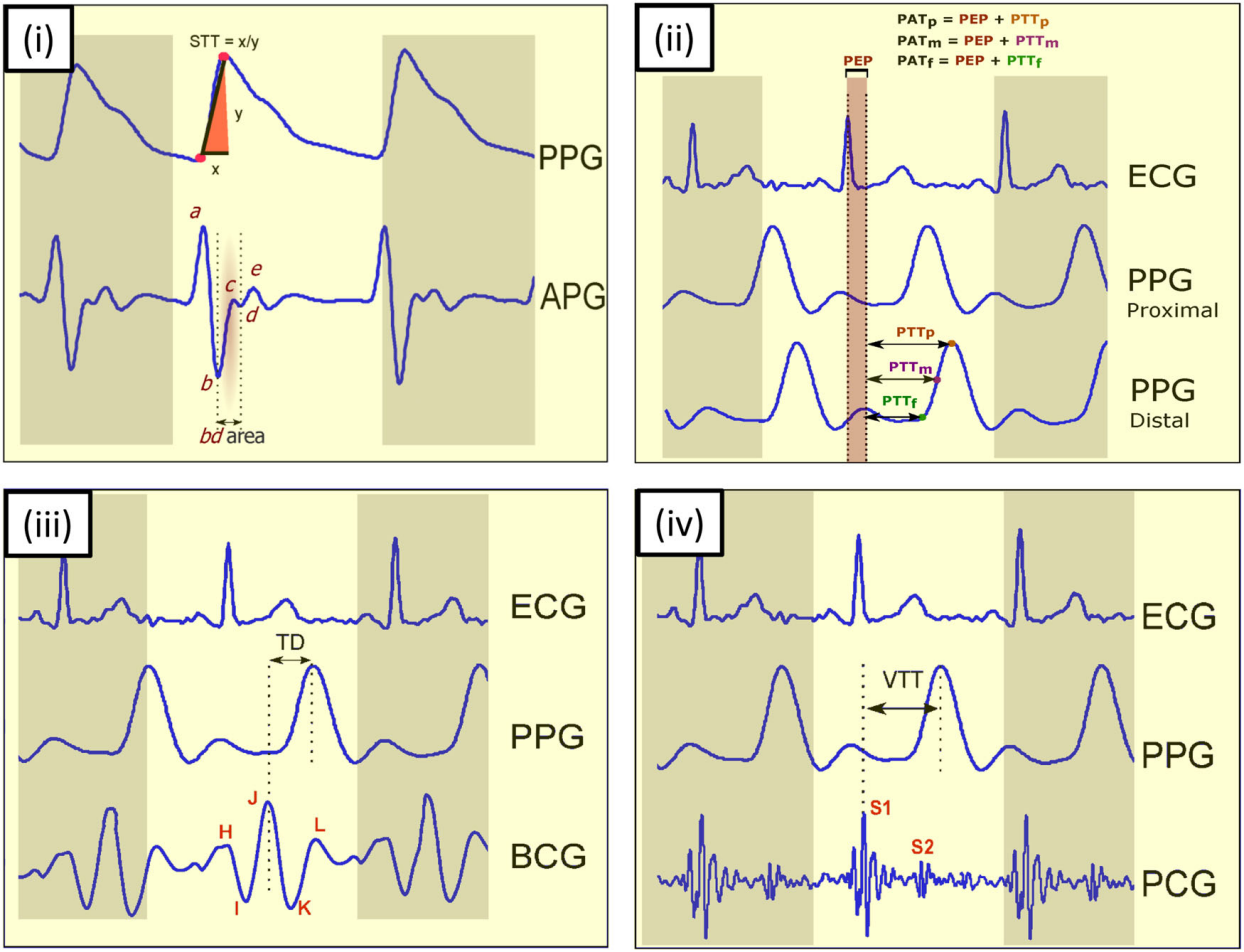
Key features of blood pressure estimation using PPG and other physiological signals. (i) Using PPG signal and its derivative, (ii) using ECG and PPG signals, (iii) using BCG signals and PPG signals, and (iv) using PCG and PPG signals. Here, *PPG* photoplethysmogram, *APG* acceleration photoplethysmogram, *BCG* ballistocardiogram, *PCG* phonocardiogram, *STT* slope transit time, *PTT* pulse transit time, *PEP* pre-ejection period, *PAT* pulse arrival time, *TD* time interval between the J peak in the BCG signal and the systolic peak in the PPG signal, *VTT* vascular time interval between the first heart sound *S*1 and the systolic peak in the PPG signal, *S*1 first heart sound, *S*2 second heart sound

In 2016, Addison^26^ found a single feature that is correlated with BP, called the slope transit time (STT) that requires only a single PPG signal. The STT reflects the steep trend of rising pulse wave. It is a slope parameter calculated from the foot to peak of the systolic waveform, as shown in Fig. 1i. In 2018, Liang et al.^27^ found that the *bd* area, shown in Fig. 1i, is also associated with BP.

## Electrocardiography and photoplethysmography

**Fig. 2:**
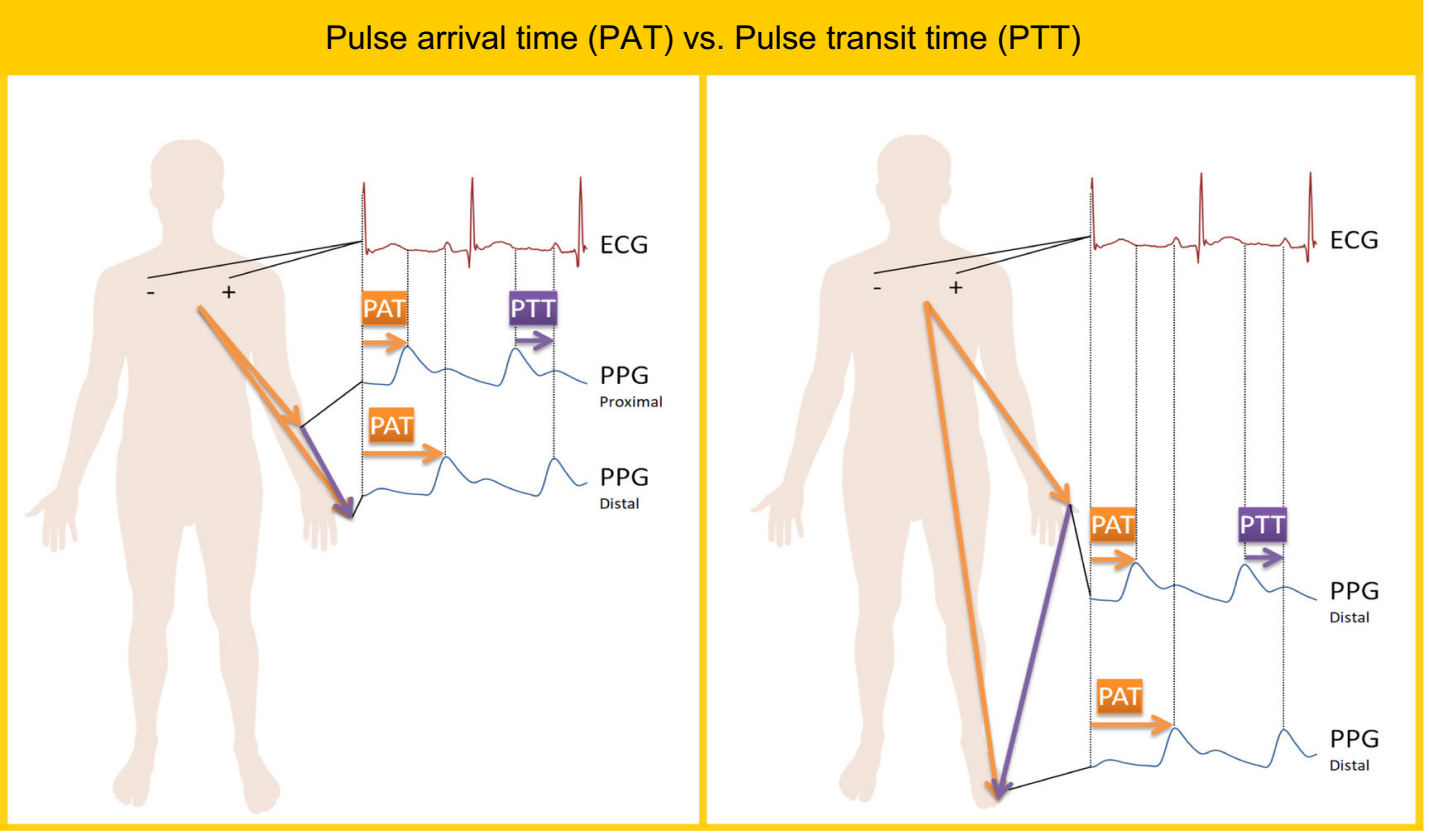
Difference between pulse arrival time (PAT) and pulse transit time (PTT). The PAT is defined as the time taken from the pulse waveform to traverse from the heart to a distal site. The PTT is defined as the period from relatively proximal site (e.g., arm) to a distal site (e.g., finger) or between two distal sites (e.g., figure and toe)

The pulse arrival time (PAT) and pulse transition time (PTT) parameters are often used interchangeably;^28^ however, these propagation times are defined differently. As shown in Fig. 1ii and Fig. 2, the PAT interval includes the PTT interval plus the pre-ejection period (PEP), which is the additional delay time between the electrical depolarization of the left ventricle (as indicated by the ECG QRS complex) and the start of the mechanical ventricular ejection. Examples shown in Fig. 2 that demonstrate calculation of PAT and PTT durations (please note, when the PTT is divided by the distance, the results is referred to as the pulse wave velocity).^29^

Although within the literature, there exists some inconsistencies with regards to the fiducial points that are used to define the start and end points for the PTT and PAT intervals, we can also find some general conventions.^28–30^ For measurement of PAT, the commonly utilized fiducial points are the R-wave of the ECG and systolic peak of the PPG waveform, which is measured at a distal site, such as the fingertip. For measurement of PTT, generally two arterial sites are used, such as the PPG proximal systolic peak waveform as measured from the upper arm, and the distal systolic peak of the PPG signal as measured from the fingertip.^28^ Interestingly, using different PPG fiducial points differentially impacts the accuracy of BP calculations.^27^

In order to avoid the variable PEP time in the estimation of blood pressure, some recent attempts have been made to determine PTT directly from multiple PPG signals. For example, Nitzan et al.^30^ determined the PTT using PPG signals measured from the toe and finger simultaneously. Note that the PTT duration was calculated from a distal site (figure) to a distal site (toe). They hypothesized that PTT measurement using PPG signals from different sites (finger and toe) would be more accurate than using PPG in combination with ECG signals. They reported that the PTT measured from the PPG_toe_ and PPG_finger_, as shown in Fig. 2, can be used as a replacement for the PTT calculated using ECG and PPG_toe_; however, the PTT calculated using ECG and PPG_toe_ was found to have a better correlation with SBP than the PTT calculated from PPG_toe_ and PPG_finger_.

## Ballistocardiography and photoplethysmography

Ballistocardiography (BCG) is a long-established technique for evaluating the cardiovascular health of patients that records the vibrations produced in the body during the cardiac cycle. Unlike the measurement of ECG signals, BCG does not require the skin contact and can easily estimate heartbeat information from patients, which makes it suitable for long-term monitoring and measurement.^31^ Each BCG beat consists mainly of five waves: H, I, J, K, and L waves,^32^ which were clearly identified to represent heart activity information, as shown in Fig. 1iii. Chen et al.^33^ found that the time difference (TD) between predetermined BCG indicia (e.g., J peak in BCG waveform) and predetermined PPG indicia (e.g., systolic peak in PPG waveform) correlated with systolic and diastolic blood pressure. Moreover, they used the TD to predict the subject’s blood pressure.

## Phonocardiography and photoplethysmography

Heart sounds associated with valve movement is recorded using the phonocardiograph (PCG) instrument. Collected sounds can provide information about the mechanical cardiac function and blood flow.^34^ In addition to the two main heart sounds, designated as S1 and S2, the waveform of the PCG signal also contains useful diagnostic information that can reveal abnormalities in the movement of the heart wall, closure of the valves, or leakage of blood flow.^35^ For the purpose of estimating BP, the PCG signal is often used together with the PPG signal from a distal arterial site to calculate another propagation time known as the vascular transit time (VTT).^36^ The VTT is derived from the first heart sound (known as S1) of a PCG and the systolic peak of the corresponding PPG, as shown in Fig. 1iv.

## Application of PPG in mobile and wearable health devices

With the goal of achieving long-term continuous BP estimation, many companies and academic research groups have explored various ways to measure BP with mobile phones or wearable sensors. Many challenges exist in achieving this goal; however, the clinical benefits of such technology still require further development.^37^ At present, commercial mobile and wearable devices can measure a variety of physiological parameters, including heart rate, body temperature, skin conductance, and physical activity. Adding the estimation of SBP and DBP is logical and expected.^38^ A list of wearable BP estimation devices, as well as descriptions of the devices and their functions, is presented in Table 1.

**Table 1:** A comparison between different wearable blood pressure estimation studies and devices

Year	Author	Wearable type	Sensors	Transmission mode	# Subjects	*f*	*r* (*f*,SBP)
2019	Redha et al.^56^	Wristband	PPG	N/R	*n*_1_ = 106	Feature set	0.69
2017	Holz et al.^53^	Eyeglass frame and finger probe	PPG	N/R	*n*_1_ = 4	PTT	0.64–0.84
2017	Zhang et al.^43^	Armband	ECG and PPG	USB cable	*n*_1_ = 10	PAT	N/R
2016	Plante et al.^50^	Mobile phone (camera + microphone)	Heart sound and PPG	N/R	*n*_1_ = 85	VTT	≈0.4
2016	Seeberg et al.^44^	Chest belt	ECG and PPG	Bluetooth	*n*_1_ = 16	PTT	−0.56
2016	Griggs et al.^42^	Bicep- and wrist-worn device	ECG and PPG	Radio frequency	*n*_1_ = 8	PAT	−0.7
2016	Zheng et al.^17^	Armband	ECG and PPG	Bluetooth	*n*_1_ = 9, *n*_2_ = 15	PAT	N/R
2015	Munnoch and Jiang^79^	Handheld	ECG and PPG	Bluetooth	*n*_1_ = 2	PAT	N/R
2014	Jung et al.^39^	Finger probe and chest pad	ECG and PPG	Bluetooth	N/R	PAT	N/R
2014	Thomas et al.^80^	Wrist watch	ECG and PPG	Bluetooth	N/R	PAT	−0.55
2012	Miao et al.^81^	Portable device	ECG and PPG	Bluetooth	N/R	N/R	N/R
2009	Guo et al.^40^	Wrist watch and finger probe	ECG and PPG	ZigBee	N/R	PAT	N/R
2008	Pandian et al.^45^	Vest-worn device	ECG and PPG	Radio frequency	*n*_1_ = 25	PAT	N/R

*r* Pearson’s correlation coefficient, *f* PPG-based feature(s), *N/R* not reported, *n*_1_ number of healthy subjects, *n*_2_ number of hypertensive subjects, *PAT* pulse arrival time, *PTT* pulse transit time, *VTT* vascular transit time

As shown in Table 1, the usual wearable and portable devices are the finger probe,^39,40^ wristband,^40–42^ armband,^17,43^ chest belt^44^, and vest.^45^ Bluetooth and ZigBee are the most utilized transmission mode.

## Pulse wave analysis methods

Before the emergence of smartphones, a variety of wearable devices were developed that used PPG sensors and PPG signal analysis to estimate blood pressure. One important consideration is that the PPG signal amplitude critically depends on the applied external pressure as well as the hydrostatic pressure, which is determined by the relative height of the PPG measurement site with respect to the heart. Several research groups have explored this relationship in order to estimate BP using a method analogous to the oscillometric method used in BP cuffs.

Instead of varying the externally applied pressure to perform an oscillometric BP measurement, a clever method was demonstrated a few years earlier in 2007 by Shaltis et al.^46^ to perform an oscillometric measurement using PPG by varying the hydrostatic pressure. This method was primarily demonstrated using a device in the form of a wearable ring, which measured PPG on the user’s finger, and contained a 3-axis accelerometer used to measure the orientation of the hand and arm. By raising and lowering the arm, it was possible to vary the hydrostatic pressure over enough range to be able to vary the PPG amplitude and thus estimate the mean arterial pressure. While such methods may be difficult to implement in practice, this research demonstrated that it is possible to create oscillometric methods to measure BP which do not require a cuff or significant electric power.

Shortly after the emergence of the conventional smartphone in 2007 (iPhone) and 2008 (Android), it was soon discovered that the smartphone camera could be used as a photoplethysmographic sensor to obtain a PPG waveform.^47^ Since 2011, several companies, such as Azumio Inc. (Palo Alto, CA, USA) began to release mobile apps that make use of this technique to measure heart rate and heart rate variability, with more recent efforts using the PPG modality to target-specific heart diseases, such as atrial fibrillation.^48^ Using the PPG signal derived from the phone camera, oscillometric methods have also been attempted. However, when a pressure cuff is not used, the main technical challenge is determining the exact pressure applied by a finger on the phone camera.

The iCare Health Monitor, released in 2016 by iCareFit Studio (http://www.icarefit.com/), makes use of the smartphone camera to acquire a finger PPG signal and estimate the BP. Although iCareFit does not disclose its exact method for deriving BP from the PPG signal, the mobile app instructions require the user to press one finger on the mobile phone camera, and simultaneously press the opposing thumb on the front touch screen of the phone, which balances the force of the finger pressing against the back-side camera. Using the touch screen sensor to estimate the finger pressure, it is possible to implement the oscillometric measurement method for BP.

A true oscillometric method was recently developed by Chandrasekhar,^2^ which uses an external pressure sensor and external PPG sensor embedded inside a custom phone case that communicates with the phone over a Bluetooth connection. Although calibration with a conventional BP cuff is required, an acceptable performance was achieved. Using visual guidance from the mobile app, users were able to learn the finger actuation required by the smartphone-based device after one or two practice trials, and subsequently, bias and precision errors of 3.3 and 8.8 mmHg for systolic BP were obtained, which achieved the recommendation of the Association for the Advancement of Medical Instrumentation (AAMI).

## Wave propagation methods

Wearable and smartphone devices have also used wave propagation methods to estimate BP using PPG signals. For example, in 2007, McCombie et al.^29^ demonstrated a wearable device to measure PTT, which consisted of a PPG wristband combined with a PPG ring. By measuring the PTT from the two sites on the hand, it was possible to estimate blood pressure using an adaptive algorithm that makes use of the Moens–Kortweg equation and a nonlinear mechanical model of the arterial walls, as follows:

1 $${\rm BP} = \frac{{K_1}}{{\rm PTT}} + K_2,$$

where *K*_1_ and *K*_2_ are calibration constants.

The estimated BP values were compared with the continuous BP values as measured by a commercial Finapres device from the same hand, and the results had good qualitative although not quantitative agreement.

In 2013, Chandrasekaran et al.^49^ demonstrated the measurement of BP using the PPG signal collected using the phone camera, in conjunction with the heart sound waveform collected using the mobile phone microphone. Using these two measurements, it was possible to calculate the VTT (time between the S1 wave in heart sound and systolic peak in PPG, described previously), and subsequently use this calculation to estimate BP. The following year, AuraLife (Newport Beach, CA) released the first commercial mobile app, named Instant Blood Pressure (IBP), which used this method to measure BP. While this approach seems promising, the specific implementation in the IBP app demonstrated poor performance when clinically tested.^50^ While Aura Labs never published any validation study for their app, an independent investigation was performed by Plante et al.^50^ The findings of this clinical study showed unreliable BP estimations and weak correlation between the estimated BP using the app and the cuff-based BP readings. Significant concerns were raised from the clinical community that individuals may use these apps to assess their BP, and the app was subsequently removed from the Apple App store.

Also in 2014, Azoi Inc.^51^ (San Francisco, USA) released a custom case and mobile app for the iPhone, called Wello, which implemented estimation of BP using the wave propagation method. The raw signals were provided by electronic sensors embedded in the custom phone case, which included an ECG sensor as well as two PPG sensors. By holding the phone with both hands, the mobile app was able to capture two measurements of PAT and use that to estimate BP. Up to our knowledge, there is no clinical study validates the use of this product.

More recently, Wang et al.^52^ demonstrated a similar mobile app which uses the PTT measurement derived from the seismic waveform of the user’s heart as measured by the phone’s internal accelerometer. Blood pressure estimates were computed using the familiar Moens–Kortweg simplification using PTT, as described in Eq. (1). Wang et al. reported good agreement between the measured BP value and the diastolic BP value measured by an external commercial BP cuff device, with an error range of ±6.7 mmHg.

In 2017, Holz et al.^53^ demonstrated that the design of eyeglass frames with embedded PPG sensors is suitable for BP measurement. This prototype device, Glabella (Microsoft Research, USA), makes use of three miniature PPG sensors that sample the pulse waveform at three different locations: the angular artery near the bridge of the nose, the temporal artery on the side of the head, and the occipital artery behind the ear. Relative blood pressure was calculated by measurement of PTT by measuring the pulse time from the angular artery to one of the other two locations, with the temporal artery yielding slightly better results. PPG data were collected from only four participants over a period of 5 days (12 + h per day) along with three blood pressure measurements per hour using an oscillometric BP cuff device, and the data were fit to the linear BP estimation equation using PTT (Eq. (1)). Using a baseline calibration, the predicted systolic BP value was found to correlate with the measured systolic BP reasonably well, with a correlation coefficient of *r* *=* 0.79 with an error of ±10 mmHg. To the best of our knowledge, there is no clinical study that validates the use of this product.

**Fig. 3:**
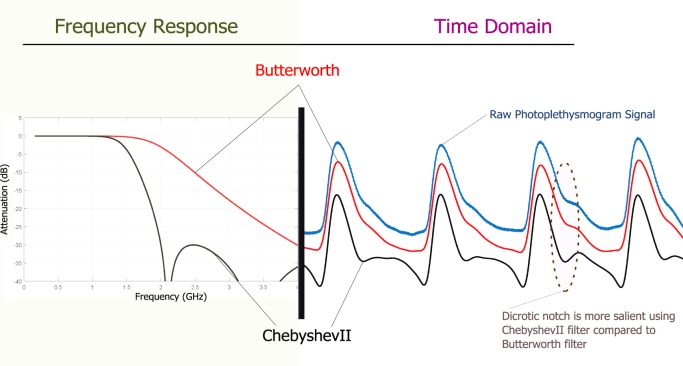
Filter impact on PPG morphology. The left figure shows the impulse response difference between the Butterworth (red line) and ChebyshevII (black line) filters. The right figure shows the Butterworth bandpass filtered (red line) and the ChebyshevII bandpass (black line) filtered PPG signals of the raw PPG signal (blue line). It is clear that the ChebyshevII filter is able to emphasize the difference between the systolic and diastolic waves, compared to the Butterworth filter.^6^ *PPG* photoplethysmogram, *dB* Decibel, *GHz* Gigahertz

It is worth noting that designing an efficient filter plays an important role in processing PPG signals. In addition, filtering algorithms can also produce time-shifts in the position of time series features. In much of the literature, filtering of the PPG time series data is common practice; however, the choice of filter is not often discussed. Butterworth filters are particularly common for filtering PPG data.^54–57^ A comparative filter study^6^ was conducted and an optimal filter for short-term PPG signal was achieved. The conclusion of this large study is that for a short duration (2 s) PPG signal, the ChebyshevII filter is more efficient for making the dicrotic notch more salient, compared with the Butterworth filter, as shown in Fig. 3. The Butterworth filter is a maximally flat magnitude filter that rolls off more slowly without ripples around the cutoff frequency, compared with other filters, such as the Chebyshev filter. When applied to a PPG time series, such filtering may lead to the disappearance of the dicrotic notch, especially if the raw PPG waveform is noisy. However, the ChebyshevII filter is able to emphasize the difference between the systolic and diastolic waves, making the dicrotic notch more visible, easy to detect, and prepares the PPG morphology for an analysis, as shown in Fig. 3.

## Models and machine learning

New types of data analysis methods have also emerged over the past decade, and this has also been applied to the problem of BP estimation derived from the raw PPG signals. In 2014, Choudhury et al.^58^ demonstrated how PPG parameters could be fit to a parametric Windkessel model to estimate blood pressure. The Windkessel model describes the arterial blood flow as an electric circuit that contains two elements: resistance and capacity.^59^ Choudhury et al. used linear regression to derive the resistive and capacitive elements for a 2-element Windkessel model, and was able to achieve moderate agreement, using PPG and BP data collected from resting hospital patients. Banerjee et al.^60^ subsequently demonstrated the use of multi-layer neural networks to derive the resistive and capacitive element values for the 2-element Windkessel model using the PPG data collected from a smartphone. The authors claimed the resulting mobile app, named HeartSense, produced results within ±10% of sphygmomanometer readings; however, due to insufficient clinical validation, as of 2018, the mobile app is no longer available in the Google Play store, and no additional information is available.

With the increasing popularity of machine-learning methods, other research has continued to explore methods of estimating blood pressure using features of the PPG signal alone without the aid of any additional sensors. In 2016, Sola et al. demonstrated the estimation of BP from the finger PPG signal using standard machine-learning and linear regression.^61^ By including PPG features (not explicitly stated the features used), they were able to predict BP with an error less than 8 mmHg. We note that the published study was conducted with only three subjects, and larger published studies are not readily available.

Following a similar approach, in 2018, Radha et al. demonstrated the use of machine-learning neural nets to estimate BP using a variety of PPG signal features, including time-domain, frequency-domain, and entropy-based features.^41^ This neural net approach was able to achieve an error of ±9.82 mmHg and ±3.88 mmHg for SBP and DBP, respectively, with Pearson correlation coefficients of 0.68 and 0.74, respectively.

A recent PPG-based machine-learning study,^4^ which used the GoogLeNet pretrained convolutional neural network, provided similar or slightly better accuracy for hypertension stratification, compared with the traditional feature extraction approach over the same data set. Thus, the use of deep learning in hypertension assessment using PPG signals is promising and may evolve over time. However, it does not yet provide clinical insights as can be seen with the traditional feature extraction followed by classical machine-learning algorithms.

## Commercialization

The use of PPG to estimate blood pressure has been demonstrated primarily by research groups or small start-up companies. However, these methods are gaining wider acceptance and mobile phone manufacturers, such as Samsung, have begun to integrate BP measurement capability into the stock mobile phone software. In 2018, in partnership with UC San Francisco, Samsung released “My BP Lab”, which is a mobile app that measures changes in blood pressure using the finger PPG signal collected from the mobile phone camera.^62^ Samsung has not disclosed the exact method used to calculate BP; however, several of the methods mentioned in this review are possible candidates. A recent review^63^ of the BP measurement app for the Samsung S9 and S9 + demonstrated good agreement with a commercial BP cuff device when the user is at rest. However, some discrepancy in the readings appeared when measured shortly after exercise.

Based on the reported sample sizes and limited validation attempts seen in publications listed in Table 1, it is difficult to confidently understand the accuracy of the BP estimations attained using PPG-based wearable devices. All studies, except one, used data collected solely from normal healthy individuals. Moreover, the sample size was very small for the majority of studies, which does not indicate reliable findings and robust analysis. To increase reliability and validity for this line of research, some recommendations will be discussed later in the Future Directions section.

## Databases availability

To the best of our knowledge, there are two main publicly available databases. The first database, called PPG-BP Database, was recently published^64^ and it contains PPG signals collected along with BP readings from patients admitted to the Guilin People’s Hospital in Guilin, China. It includes data collected from 219 subjects, aged 21–86 years, with a median age of 58 years, covering several diseases including hypertension, diabetes, cerebral infarction, and insufficient brain blood supply. The second database is the Multiparameter Intelligent Monitoring in Intensive Care (MIMIC) Database,^65^ which contains thousands of recordings of multiple physiologic signals such as arterial blood pressure (ABP), PPG signals, and respiration, with additional waveforms simultaneously collected.

While several papers^66–68^ have used the MMIC database to assess pulse transit times, it should be noted that the PPG and ECG signal data are not perfectly synchronized. Although multi-parameter hospital monitors collect various physiological parameters “simultaneously”, the electronic hardware and software filtering on the measured signals produce additional time delays (up to 500 milliseconds), which can have a significant effect on the calculation of PAT and other propagation delays.^69^ Therefore, it is not recommended to use the MIMIC database to calculate PTT or PAT.

## Discussion

The present review aimed to provide an overview of the current methods of measuring BP that are cuffless and support continuous BP estimation. In the body of literature related to pulse morphology and pulse wave analysis, there is increasing interest in using the PPG waveform to understand the formation of blood pressure. It is generally accepted that the physiological status of peripheral blood vessels, such as aging, stiffness, and compliance, can be partially expressed in terms of peripheral signal waveforms.^23^ As demonstrated by recent research, analysis of the PPG waveform can assist in understanding the underlying status of peripheral blood vessels under the influence of blood propulsion and the blood recycling process. For this reason, many parameters extracted directly from waveforms can be used to accurately evaluate vascular status. The parameters mentioned often in the literature include pulse width, augmentation index, large artery stiffness index, crest time, etc. As described previously, the waveform’s shape is influenced by the blood circulatory system. Therefore, based on the waveform propagation and waveform morphology theories, two different research directions have been formed and developed to continuously estimate BP through cuffless methods.

Based on the current literature, there is clear evidence that the fluctuations in BP are reflected in the PPG signals. Given that the exact relationships between PPG waveforms and BP are not yet clear, BP estimates are difficult to achieve by the simple fitting of models or equations. Fortunately, continued advances in machine-learning technology will continue to provide new insights into the exact relationship between the BP and PPG waveforms. Many features, such as amplitude, time span, area, ratio, spectral information, and spectral entropy information, continue to be explored. Certainly, extracting more features^70,71^ from PPG waveforms, and using these features to create new machine-learning models, will continue to be an obvious approach to the problem of BP estimation.

In the body of literature relating to wave propagation theory, physical models can now explain the process of blood pressure propagation through the human body, and this process is presented and widely recognized in the form of pressure waves and ECG, BCG, PCG, and PPG signals. However, several aspects require special attention in determining the merits of BP estimation results, such as the transmission distance, the starting and ending points of the transmission, PTT, etc. Numerous problems still exist with respect to accurate positioning of sensors, calculation of propagation distances, and the impact of the variable PEP time on the pulse wave velocity timing. While there exists a definite formulaic relationship between BP and PTT, as described above, these practical challenges have resulted in a wide variation in systolic blood pressure estimates across different studies.

Naturally, researchers in the field have conducted a series of studies in order to address these issues. To avoid the problems associated with the PEP, different cardiac signals have been utilized in studies to obtain more accurate heart valve opening times. Many studies have shown that PCG and BCG signals could be more accurate in extracting the heart valve opening time than ECG signals. Some researchers have also conducted comparative studies on the performance of BP estimation using different ending points in the PPG signal. The literature has also evidenced the application of distinct linear and nonlinear mathematical formulas to calculate BP and to obtain better approximations. The need for individual short-term or long-term calibration has also been confirmed by a body of literature as a means of improving the accuracy of BP estimation. However, various limitations in accurate BP measurement, such as repeated calibration, inconvenient measurement locations, etc., often greatly limit the practical application of theory. In addition, based on the analysis of waveform propagation theory, most of the BP estimations are focused on SBP, and DBP has no definite theoretical relationship with any parameter derived from this theory. For this reason, many experimental studies have experienced difficulties in obtaining satisfactory DBP prediction results.

**Fig. 4:**
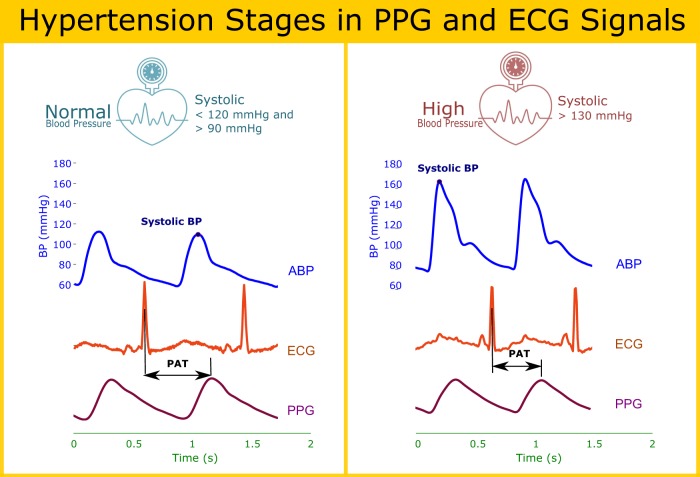
Pulse arrival time in different hypertension stages. *PAT* pulse arrival time, *ECG* electrocardiogram, *ABP* arterial blood pressure, *PPG* photoplethysmogram

Figure 4 illustrates, at a qualitative level, how PAT can be used to estimate blood pressure at different stages of hypertension. These examples were taken from the MIMIC-II database^65^ and included PPG and ECG signals, as well as the ABP which is considered the gold standard. It can be seen that there is an inverse relationship between the blood pressure and the PAT duration, the higher the blood pressure, the smaller the PAT duration, and vice versa. However, more precise correlation is not possible given that the data in MIMIC- II do not contain proper time synchronization across signals, and is thus not designed for PAT/PTT analysis.

Based on this literature review, it appears that Pearson’s correlation coefficient has been mostly adopted by researchers and is used similarly here as well. There have been two approaches for correlating PAT with blood pressure: the first approach is to use the PAT as a single feature or combined with other physiological features to classify normotensive and hypertensive subjects. The second approach is to use the PAT duration itself or combined with other features to estimate (or predict) the actual blood pressure.

Regarding the second approach, the calculation of the correlation coefficient is not enough to validate the estimated BP values. The mean error (ME) and standard deviation (STD) between predicted BP and reference BP are also needed. The estimation of blood pressure values has been standardized by the AAMI. To meet the AAMI criteria,^72^ the ME difference between the estimated BP values and the mercury standard must be ≤5 mmHg or the STD must be ≤ 8 mmHg.

At present, some studies have shown good predictive performance for continuous BP estimation techniques based on machine-learning technology, as shown in Table 2. If one feature from PPG and ECG will be used, such as PAT, the most common linear model for estimating BP is ${\mathrm{BP}} = \left( {\beta _0 \times {\mathrm{PAT}}} \right) + \beta _1$ due to its robustness to artifacts.^73^ A least square algorithm is usually implemented to determine the unknown coefficients *β*_0_ and *β*_1_, which is considered as the calibration process. If more than one feature to be used in the estimation of BP using linear regression than more coefficients need to be considered; however, there are other mathematical models can be used, as shown in Table 2.

**Table 2:** A summary of computing model and performance of the BP estimation

Year	Features	*r* (features, BP)	Model/Method	BP confidence interval in mmHg	# Subjects	Ref.
2019	10 features	*r*_s _= 0.6	*k*-nearest neighbors	N/R	*n*_1 _= 48, *n*_x _= 73	^3^
2019	PAT	*r*_s _= −0.54	BP = (*β*_0 _× PAT) + *β*_1_	N/R	*n*_1 _= 48, *n*_x _= 73	^27^
2019	N/R	*r*_s _= 0.78	Partial least-squares regression	CI_s _= −0.2.3 ± 18	*n*_1 _= 265	^82^
2018	PPG signal	N/R	Deep learning	N/R	*n*_1 _= 48, *n*_x _= 73	^4^
2016	PPG signal	N/R	Neural networks	CI_s _= 2.3 ± 2.9, CI_d _= 1.9 ± 2.5	N/R	^83^
2015	PAT, AI, LASI, IPA	N/R	Support vector machines	CI_s _= 12.3 ± 18.5, CI_d _= 6.4 ± 8.5	N/R	^75^
2014	PAT	*r*_s _= −0.67, *r*_d _= −0.61	BP = (*β*_0 _× PAT) + *β*_1_	CIs = 5.8 ± N/R, CI_d _= 5.15 ± N/R	*n*_1 _= 9	^84^
2014	PAT	N/R	BP = (*β*_0_/PAT) + *β*_1_	CI_s _= 0.1 ± 2.5, CI_d _= 1.3 ± 7.4	*n*_1 _= 30	^85^
2013	4 features	N/R	Neural networks	CI_s _= 5.2 ± 5.0, CI_d _= 2.9 ± 2.9	N/R	^86^
2013	21 features	N/R	Neural networks	CI_s _= 3.8 ± 3.5, CI_d _= 2.2 ± 2.1	N/R	^86^
2013	N/R	N/R	Neural networks	CI_s _= −2.9 ± 19.4, CI_d _= −3.7 ± 8.7	*n*_x _= 47	^87^
2013	PAT	*r*_s _= −0.71, *r*_d _= −0.69	BP = (*β*_0 _× PAT) + *β*_1_	CI_s _= 0.81 ± 5.48, CI_d _= 0.34 ± 2.94	*n*_x _= 72	^73^
2013	PAT, HR	N/R	BP = (*β*_0 _× PAT) + (*β*_1_ × HR) + *β*_2_	CI_s _= 1.8 ± N/R, CI_d _= 1.57 ± N/R	*n*_1 _= 10	^88^
2010	PTT	*r*_s _= −0.84, *r*_d _= N/R	BP = (*β*_0 _× ln(PTT)) + *β*_1_	CI_s _= N/R, CI_d _= N/R	N/R	^89^
2010	PAT, HR, TDB	N/R	BP = *β*_0 _+ (*β*_1 _× PAT) + (*β*_2_ × HR) + TDB	CI_s _= −0.002 ± 5.9, CI_d _= −0.02 ± 4.7	*n*_x _= 10	^90^
2004	PAT	N/R	BP = (*β*_0_/PAT^2^) + *β*_1_	CI_s _= 0.08 ± 11.3, CI_d _= N/R	*n*_x _= 22	^91^

*PAT* pulse arrival transit time, *PTT* pulse transit time, *HR* heart rate, *TDB* arterial stiffness index, *AI* augmentation index, *LASI* large artery stiffness index, *IPA* inflection point area ratio, *β*_0_, *β*_1_ and *β*_2_ regression coefficients, *N/R* not reported, *n*_1_ number of healthy subjects, *n*_x_ number of unhealthy subjects

CI_s_ and CI_d_ are the confidence interval (mean ± standard deviation) for the estimated systolic pressure and diastolic pressure, respectively. Here, *r*_s_ is the correlation coefficient for the systolic pressure while *r*_d_ is correlation coefficient for the diastolic pressure

It is clear from Table 2 that there is inconsistency in approaching this challenge, in terms of sample size, the number of features, reporting correlation coefficients with used feature(s), reporting the estimation error in terms ME and STD. However, despite these challenges, this line of research is encouraging.

While BP estimation from the PPG signal is relatively new, the use of PPG in the clinical setting is widespread. In the developed world, the use of the pulse oximeter for anesthesia monitoring during surgery has been the standard of care for more than 20 years,^13^ and the World Health Organization is now leading the Global Pulse Oximetry Project, which aims to make the pulse oximeter component available in every operating room in the world.^74^ This widespread use of pulse oximetry represents another possible opportunity to employ the ubiquitous pulse oximeter as a device for estimating BP.

Although the emerging algorithms for BP estimation and health monitoring are increasingly complex, the processing power of wearable and mobile technologies, as well as cloud computing have also increased dramatically over the past decade. The ability of wearable health devices to monitor BP and cardiovascular health status is becoming increasingly feasible.

At present, a series of health parameters, such as body temperature, heart rate, ECG, and PPG, can be monitored by commercial wearable products. Soon, wearable BP products will also likely be an acceptable form of health monitoring utilized by most people, and their precision, wear, ease of use, and other characteristics will continue to improve significantly.

## Future directions

The use of PPG signals as a replacement for cuff-based or invasive BP measurement is relatively new, and most PPG analysis methods are still relatively simple. A variety of estimation methods continue to be explored, including those that utilize PPG signals exclusively, and those which combine PPG with other physiological signals. As can be seen from the tables, the estimation of BP from PTT continues to be perhaps the most common approach, but generally only with very small sample sizes. With the advent of wearable devices, the real-time analysis of PPG waveforms also enables the possibility to monitor BP on a nearly continuous basis.^75–77^ In the future, the increasing adoption of wearable PPG devices will also likely play an essential role in patient care.

Our recommendations, based on a review of BP estimation using PPG signals, are as follows:

1. Whenever possible, use additional physiological cardiovascular measurements (e.g., ECG, ABP, etc.) to PPG, in order to increase accuracy;
2. When multiple cardiovascular signals are used, care must be taken to ensure time synchronization across all sensors;
3. Standard and consistent use of use measurement terminology needs to be encouraged in order to avoid confusing or misleading research results (e.g., PAT vs. PTT);
4. Standards such as AAMI need be adopted for use in estimating BP using PAT, PTT, or other propagation times;
5. For the purpose of validating and labeling data, a proper FDA-approved BP measurement device needs to be used (and calibrated regularly);
6. Additional research is required using sample sizes of *n* > 100 subjects, with a mixture of both normotensive and hypertensive subjects;
7. Widespread use of BP estimation algorithms will also require additional studies that include socioeconomic diversity (ages, race, gender, etc.);
8. The robustness of BP estimation algorithms for ambulatory devices needs to be tested under a variety of movement conditions, not just sedentary;
9. The research community would benefit if published studies also included additional clinical PPG features^78^ to examine correlation with BP;
10. There is a need for more publicly available physiological databases, in addition to PPG-BP^64^ and MIMIC-II^65^ that contain time-synchronized physiological signals for the purpose of calculating PTT and PAT; and
11. Increased collaboration between engineering and clinical researchers would help enrich the validation and protocol process, in addition to helping with access to patients, and improving the quality and availability of research data.

## Conclusion

Given the widespread use of blood pressure in medicine and health care, we have provided a review of photoplethysmography as a tool for cuffless estimation of BP, examining the use of PPG independently, as well as in combination with other cardiovascular measurements. The increasing demand for the PPG-based wearable devices also provides an interesting direction for continuous ambulatory measurement of BP.

Currently, most PPG-based BP estimation is mainly divided into two research directions based on waveform morphology theory and waveform propagation theory. Research based on the waveform morphology theory, also known as pulse wave analysis, has typically relied on parameters extracted from a single PPG waveform. Some of these explorations have led to excellent research findings that have enabled the implementation of wearable designs, but many problems are present, such as the need for a large amount of data and a certain period of pre- training and calibration. Research based on the waveform propagation theory, utilizing the pulse propagation delays between multiple pulse signals, has also shown promising results. Even so, significant problems remain, relating to the acquisition of multiple signals, the location of sensors on the body, and individual factors, which can directly affect the accuracy of BP estimations. The existence of such practical problems and the difficulty in overcoming them has made the applications of this theory difficult.

The development of noninvasive, cuffless, and continuous BP estimation is a promising yet challenging field. That the trend is toward wearable BP technology is evident. In future studies, a more comprehensive understanding of PPG information can hopefully enable researchers to solve the abovementioned problems and to successfully develop technologies for BP estimation using mobile and wearable devices.
